# Nicotinamide Improves Functional Recovery via Regulation of the RAGE/JNK/NF-κB Signaling Pathway after Brain Injury

**DOI:** 10.3390/jcm8020271

**Published:** 2019-02-22

**Authors:** Sayed Ibrar Alam, Shafiq Ur Rehman, Myeong Ok Kim

**Affiliations:** Division of Life Science and Applied Life Sciences (BK21), College of Natural Sciences, Gyeongsang National University, Jinju 52828, Korea; ibrar@gnu.ac.kr (S.I.A.); Shafiq12@gnu.ac.kr (S.U.R.)

**Keywords:** brain injury, nicotinamide, neurodegeneration, synaptic dysfunction, neuroinflammation

## Abstract

Brain injuries are a serious global health issue and are the leading cause of neurodegeneration. To date, there is no proper cure and treatment for brain-injury-induced neuropathological conditions because of a lack of sufficient knowledge and the failure to develop a drug due to the multi-pathological conditions in the brain. Herein, we explored the neurotherapeutic effects of Nicotinamide (NAM), against brain injury-induced neurodegeneration and behavioral problems. Treating injured mouse brains with NAM, for 7 days, significantly ameliorated several pathological events. Interestingly, NAM treatment significantly inhibited the injury-induced activation of receptor for advanced glycation end-products (RAGE), c-Jun N-terminal kinases (JNK), and neuroinflammatory mediators, such as NF-κB, TNF-α, IL-1β, and NOS2 in the brain, and it also regulated the levels of apoptotic markers, including Bax, caspase-3, and Bcl-2. Furthermore, treatment using NAM in TBI mice, significantly reversed synaptic protein loss and improved memory impairments and behavioral outcomes. Our findings suggested that NAM treatment reduced injury-induced secondary neurodegenerative pathology by modulating RAGE/JNK/NF-κB signaling in mice. Therefore, we recommend that NAM would be a safe and efficient therapeutic agent against brain-injury-induced neurodegeneration.

## 1. Introduction

Brain injury is a major medical and socioeconomic problem and it is the leading cause of deaths among individuals involved in vehicle accidents, sports, as well as in war-affected military personnel [1,2]. The affected brain suffers structural and functional changes with a severe loss of neurons [3,4]. Brain injury is categorized as mild to severe, based on clinical indications and pathological lesions involving edema, contusion, and hemorrhage, which are considered to be the traditional clinical indicators that follow a head injury [5]. Clinical symptoms that follow a brain injury, comprise headaches, consciousness, attention problems, and memory and motor deficits [6]. Previous studies have demonstrated that survivors of brain injury develop several pathological processes, which include damage to the brain, aggression, neurological deficits, anxiety, depression, and problems associated with behavior and emotions [7,8]. Several lines of investigation have demonstrated that a possible role of chronic brain injuries that lead to secondary injuries, are related to those seen in neurodegenerative diseases, such as Alzheimer disease (AD) and Parkinson’s disease (PD) [9,10,11]. Mounting evidence in animal models of brain injury have demonstrated injury-induced changes in cognitive ability, and the accumulation of Amyloid beta (Aβ) and hyperphosphorylated tau (p-tau) proteins [12,13]. 

Other studies provide evidence that chronic brain injury is a pathological process with an initial injury that leads to several biochemical and cellular changes, which eventually lead to multiple apoptotic and inflammatory cascades in the brain, finally causing a neuronal cell death [14]. Further studies have claimed that injury to the brain causes the activation of microglia and astrocytes, blood brain barrier disruption, and upregulation of inflammatory mediators, such as TNF-α and interleukins, which further deteriorate the neuronal structure and function [15]. Both experimental and clinical reports have strongly associated pos- brain-injury consequences with persistent neurodegenerative conditions and even permanent disability [16,17]. Oxidative stress and neuroinflammation are the major hallmarks of post-brain injury consequences and neuropsychiatric disturbances [18]. Excessive reactive oxygen species (ROS) in the brain can cause damage to the enzymes, nucleic acid, and disruption of structural protein [19,20,21]. Several lines of studies have demonstrated elevated ROS and neuroinflammation in brains after brain injuries [22,23]. However, pharmacologically targeting these mediators could improve neuropsychiatric symptoms and behavioral outcomes.

The receptor for advanced glycation end-products (RAGE), which expresses on the surfaces of neurons, endothelial cells, microglia, and immune cells, is a multi-ligand receptor that binds advanced glycation end products (AGE) and beta-amyloid (Aβ). This receptor–ligand interaction activates the receptor and contributes to neuroinflammation in the brain [24,25]. The elevated expression level of RAGE was investigated in Alzheimer’s disease and in an aging mouse model [25]. Studies reported that activation of RAGE triggers a variety of signaling cascades for immune responses, which include the activation of NF-κB and the further activation of pro-inflammatory cytokines generation [26,27]. C-Jun N-terminal kinases (JNKs) play an essential role in neuronal plasticity, regeneration, and cell death, via the stress signaling pathway, and its activation has a fundamental rule in cell apoptosis and inflammation [28].

Nicotinamide is the amide form of niacin and it is an important precursor of Nicotinamide adenine dinucleotide (NAD), which is required for energy metabolism and cellular functions [29]. An oxidized form of Nicotinamide (NAD^+^) is necessary for mitochondrial enzyme reactions and cellular energy metabolism [30]. Nicotinamide provides a broad range of neuroprotective effects against several stimuli, such as oxidative stress, free radical generation, strokes, and cerebral ischemia [29,31]. The protective effect of Nicotinamide has shown that it can inhibit poly-adenosine diphosphosphate-ribose polymerase-1 (PARP-1) and the activation of sirtuins-1(SIRT-1), after a brain injury [32]. Overcoming NAD^+^ depletion and regulation of cellular energy deficits would be a therapeutic target for the prevention of neurodegenerative disease.

Nicotinamide neuroprotection in brain injuries has not been broadly explored. In this study, we have developed a cortical stab wound injury in a model mouse that exhibited chronic neuroinflammation and oxidative stress. The model mouse was treated using Nicotinamide (NAM) to explore its therapeutic potential in brain injuries. Administration of NAM ameliorated RAGE-mediated neuroinflammation, and oxidative stress-induced neurodegeneration was performed in the mice model. Taken together, the data showed that administration of NAM ameliorates neuropathologies in an animal model of brain injury, via regulation of the RAGE/JNK/NF-κB signaling. Our study suggests that NAM has a disease-modifying therapeutic potential in injury-induced neurodegeneration. 

## 2. Materials and Methods

### 2.1. Animals

Male C57BL/6N mice (10 weeks; average body weight 30 g) were obtained from Samtako Bio, Korea. All animals were maintained in the departmental animal house for acclimatization at 23 °C temperature, with 60 ± 10% humidity, under a 12/12 h light/dark cycle, and were provided with food and water, ad libitum. The experimental procedures were conducted, according to the animal ethics committee (IACUC) of the Division of Applied Life Science, Department of Biology, Gyeongsang National University, South Korea (Approval ID: 125). Nicotinamide (Sigma, St. Louis, MO, USA) was dissolved in saline and administered to the brain injured animals, using a daily intraperitoneal (i.p.) injection, for 7 days. 

### 2.2. Stab Wound Cortical Injury (SWI)

The stab wound cortical injury mice model was prepared, as previously described in References [33,34], including the modifications. The animals were randomly divided into two groups and were subjected to either stab wound cortical brain injury (SWI) or stab wound cortical brain injury plus NAM (SWI + NAM) treatment (*n* = 13). Briefly, the animals were anesthetized with an intraperitoneal injection of Zoletil and Rompun, followed by a longitudinal incision, to expose the skull. The unilateral craniotomy, 4 mm in diameter, was performed (2 mm lateral to the midline and 1.5 mm posterior to the bregma), using a dental drill. Producing a stab wound cortical injury, a sharp edge scalpel blade was inserted 3 mm into the right hemisphere of the brain. The scalpel blade remained in the cortex for 1 min and was slowly removed. The skull was covered with bone wax and the skin was closed with a silk suture. All animals were visually monitored, until their safe recovery from anesthesia. 

### 2.3. Treatment 

**Figure 1:**
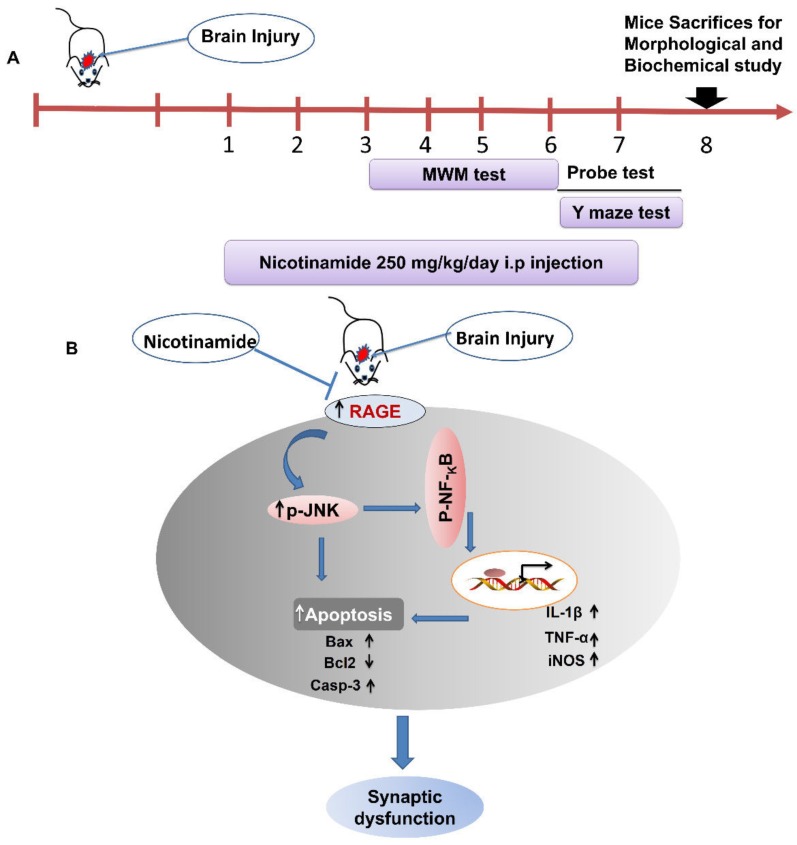
The schematic diagram represents the treatment schedule and the mechanism of NAM neuroprotection in mouse brains. The schematic representation (**A**) showing that NAM was treated for 7 days following the brain injury in mice and (**B**) showing that NAM treatment for 7 days ameliorated neuroinflammation, neuronal apoptosis, and rescued memory impairment via regulation of RAGE/JNK/NF-KB signaling pathway after mouse brain injury.

For treatment, the mice were selected and classified into four groups: control saline-treated, stab wound cortical injury (SWI), stab wound cortical injury plus NAM (SWI + NAM), and sham-treated group (NAM). For treatment, 250 mg/kg of NAM was dissolved in distilled water and was administrated via a daily intraperitoneal injection, for 1 week. NAM treatment was started 1 h later when the animals were fully recovered from the anesthesia. The treatment schedule of the NAM in brain injury mouse model is explained in Figure 1A

### 2.4. Morris Water Maze (MWM) Test 

For behavior analysis, the animals were allowed in the MWM tank for habituation. After three days, following injury and NAM treatment, all mice were brought into the MWM to check the cognitive ability of the treated mice. Behavior analysis was performed as reported earlier in Reference [35]. The apparatus was composed of a circular tank filled with water and made opaque with white ink, with a hidden platform. The data were recorded with the help of a video tracking system (SMART, Panlab Harvard Apparatus, Bioscience Company, Holliston, MA, USA). The behavior study was performed for 4 consecutive days. The mice were subjected to training for 3 trials per day, followed by a probe test on day 5, when the hidden platform was removed. The latency time, the number of crossings, and the time spent in the target quadrant was recorded. Following the behavior analysis, the mice were killed and processed for further immunoblot and immunohistological analysis. 

### 2.5. Y-maze Test

The black-pointed Y-maze was used for spontaneous alternation behavior of the treated groups. The length of each arm was 50 cm long and had a 10 cm width, at the top and bottom. The mice were allowed into the center of the apparatus and were able to move freely in each arm, for three 8 min sessions. The entries of the mice to each arm were noted. The spontaneous alternation was defined as the successive entry of the mice into the three arms, in overlapping triplet sets. Alternation behavior (%) was calculated using the formula—successive triplet sets divided by the total number of arms entrie minus 2 × 100.

### 2.6. Beam Walking Test

The beam walking test was performed, as previously demonstrated in Reference [36], with modifications. The beam walking test is commonly used to analyze fine motor coordination among the different treatment groups. The data were analyzed on different days, following brain injury.

### 2.7. Protein Extraction from Brain 

All mice were first anesthetized and sacrificed immediately after the behavior analysis. First, we collected each brain and then the cortex and hippocampus tissue were carefully dissected. Each tissue was frozen and stored at −80 °C, followed by homogenization in PRO-PREP^TM^ solution (iNtRON Biotechnology, Burlington, NJ, USA), for a further biochemical analysis.

### 2.8. Western Blot Analysis

The protein expression level was examined, as previously described in References [37,38,39], with minor changes. Protein was separated by gel electrophoresis and was transferred onto a Polyvinylidene difluoride (PVDF) membrane, followed by blocking in a 5% skim milk solution. The membrane was incubated with primary antibodies to detect the different protein expression levels. The primary antibodies were allowed for attachment, overnight, at 4 °C. Next day, the membranes were incubated with horseradish peroxidase (HRP) conjugated secondary antibodies for 1 h. The membranes were then developed in a dark room, using ECL chemiluminescence (Atto Corporation Tokyo, Japan).

### 2.9. Tissue Collection and Sample Preparation

For immunofluorescence and histological analysis, as described previously in Reference [40,41], the mice were transcardially perfused with saline, followed by fixation with 4% ice-cold paraformaldehyde, and the brains were fixed for 72 h, in paraformaldehyde, and then transferred to 20% sucrose, for 72 h. The O.C.T compound (tissue-Tek O.C.T compound medium, Sakura Finetek USA, Inc., Torrance, CA, USA) was used for freezing and blocking the brain, and 14 μm coronal sections were cut, using a CM 3050C cryostat (Leica, Nussloch, Germany). The brain section was taken on a probe-on plus charged slides (Fisher, Rock-ford, IL, USA). 

### 2.10. Assessment of Lesion Volume 

To analyze the lesion volume following brain injury, the tissues on the slides were stained with cresyl violet, according to the previous method, with minor modifications [36].

### 2.11. Immunofluorescence Staining 

Immunofluorescence staining was performed as previously described, with a modification [42,43]. Initially, the slides were dried overnight before staining, followed by washing with PBS (0.01 Mm), twice, for 10 min. The slides were incubated with proteinase K for 5 min and then rinsed with PBS (0.01Mm). For blocking, 2% normal serum was applied (goat/rabbit) with 0.1% Triton X-100 in PBS. After blocking, the slides were carefully incubated with primary antibodies, overnight, including Glial fibrillary acidic protein (GFAP), Caspase-3, p-JNK, IL-1β Iba-1, PSD-95, and Synaptophysin. The secondary antibodies, Tetramethylrhodamine (TRITC) and Fluorescein isothiocyanate (FITC)-labeled (1:100), were incubated for 90 min, followed by 4′,6-diamidino-2-phenylindole (DAPI) for the nucleus detection. The slides were covered using coverslips with a mounting medium, and images were taken through a confocal laser-scanning microscope (Flouview FV 1000 MPE, Olympus, Japan ). For the quantification of the amount of staining in the fluorescence images obtained with confocal microscopy, Integrated Density was used. Integrated Density was determined using ImageJ software (version 1.50, NIH, https://imagej.nih.gov/ij/, USA) and it represents the sum of the values of the pixels in an image. 

### 2.12. Nissl Staining 

To analyze the extent of neuronal cell death, Nissel staining was performed, as previously described, with minor modifications [44]. Briefly, the slides were washed with 0.5% cresyl violet solution for 10–15 min. Furthermore, the slides were washed with distilled water and were dehydrated in graded ethanol series (70%, 95%, and 100%), then mounted with the mounting medium, covered with coverslips, and examined with a light microscope.

### 2.13. Fluoro-Jade B (FJB) Staining 

Fluoro-jade B staining was performed, as previously described [45,46]. Briefly, the tissue slides were washed twice, for 5 min, in 0.01M PBS and then immersed in a solution of 1% sodium hydroxide and 80% ethanol, for 5 min. The slides were then immersed in 70% alcohol and distilled water for 2 min, respectively. Moreover, the slides were kept for 10 min in 0.06% MgCl_2_ solution in the dark, followed by slow shaking, and then washed with distilled water for 5 min. Finally, the slides were transferred to the solution containing acetic acid (0.1%) and an FJB (0.01%) solution, for 12 min. The slides were then cleared with xylene and mounted with the mounting medium (Dako Fluorescence Mounting Medium, Carpinteria, CA, USA). DAPI was used for nucleus detection and the glasses were covered with slips. Images were obtained using confocal laser microscopy (Flouview FV 1000, Olympus, Japan).

### 2.14. Statistical Aanalysis

The immunoblot band was scanned and analyzed using sigma Gel software (version 1.0, SPSS, Inc., Chicago, IL, USA). ImageJ Software was used for the immunohistological quantitative analysis. One-way ANOVA followed by post hoc analysis was used. Statistical analyses were performed using Graph-Pad Prism 5 software. The values were the mean ± S.E.M. *P* value less than 0.05 was considered statistically significant. The symbols * *P* < 0.05 and ** *P* < 0.01 indicates the significant differences between saline-treated and SWI; # *P* < 0.05 and ## *P* < 0.01 indicates significance differences between SWI and SWI plus NAM. 

## 3. Results

### 3.1. NAM Ameliorates RAGE, JNK, and NF-ΚB Signaling in Injured Mouse Brains

**Figure 2:**
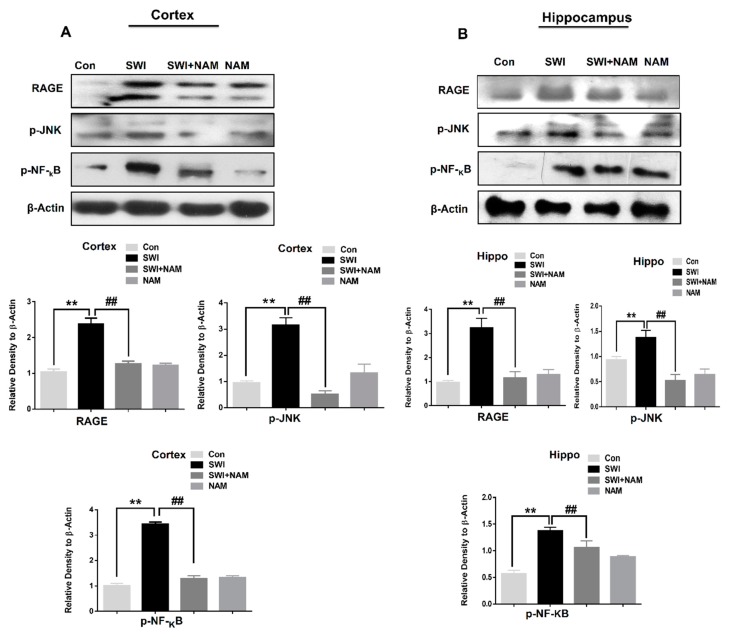
Nicotinamide (NAM) attenuates the expression level of receptor for advanced glycation end-products (RAGE), p-JNK, and p-NF-κB in the mouse brains. (**A,B**). Representatives immunoblots and histograms showing the expression level of RAGE, P-Jun N-terminal kinases (JNK), and p-NF-κB in the ipsilateral cortex and hippocampus region of the brain injury and brain injury plus treated groups (*n* = 8). The β-actin was used as a loading control. The sigma gel software was used for the quantification of protein bands. The values are the mean ± SEM. a *P*-value less than 0.05 was considered significant. All the values were taken from three independent experiments. One-way ANOVA followed by post hoc analysis for the comparison of the values. *P* < 0.05 was considered statistically significant. The symbols ** *P* < 0.01 represent significant differences between control and SWI; ## *P* < 0.01 represent significance differences between SWI and SWI plus NAM.

The activation of RAGE and oxidative stress is involved in several neurodegenerative diseases [47]. We further extended our line of investigation and examined the expression level of RAGE and the mechanism of NAM neuroprotection in the brain injured animal model, at day 7. Our data showed the increased expression levels of RAGE, p-JNK, and p-NF-_K_B in the ipsilateral sides of the cortex and hippocampus of the injured mouse brains, compared to the saline-treated mice group, at day 7. However, the treatment using NAM significantly reduced the levels of RAGE, F _0.05(__3, 8)_ = 11.50; *P* < 0.01, p-JNK, F _0.05__(3, 8)_ = 11.80; *P* < 0.01, and p-NF-_K_B, F _0.05(__3, 8)_ = 17.41; *P* < 0.01, in the ipsilateral cortex and the hippocampus [RAGE, F _0.05(__3, 8)_ = 15.42; *P* < 0.01, p-JNK, F _0.05__(3, 8)_ = 17.10; *P* < 0.01, and p-NF-_K_B, F _0.05__(3, 8)_ = 17.88; *P* < 0.01] of the injured mouse brains (Figure 2).

**Figure 3:**
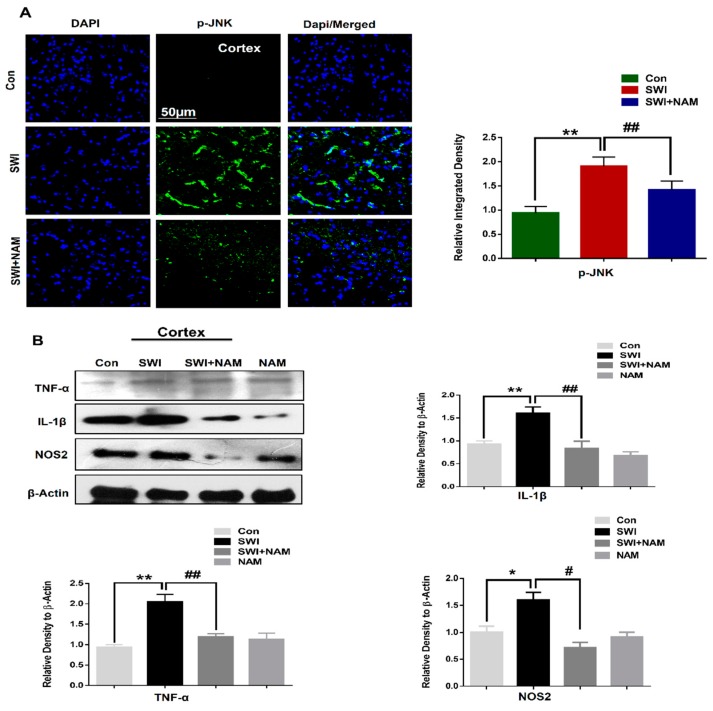
NAM treatment reduced activated JNK and expression level of inflammatory markers in the injured mouse brains. (**A**) The confocal images of p-JNK among the treated mice group, at day 7 (*n* = 7). (**B**) The immunoblots and histograms represent the expression levels of pro-inflammatory markers, such as tumor necrosis factor-alpha (TNFα), interleukin-1 beta (IL1-β), and nitric oxide synthase 2( NOS-2) among the treated groups at day 7 (*n* = 8). The blots were quantified using sigma gel software. β-actin was applied as a loading control. The ImageJ software was used for histological analysis (Magnification X10). All values were taken from three independent experiments. One-way ANOVA was done, followed by post hoc analysis for the comparison of values. *P* < 0.05 was considered to be statistically significant. The symbols * *P* < 0.05, and ** *P* < 0.01 represent significant differences between the control and the SWI; # *P* < 0.05, and ## *P* < 0.01 represent significance differences between the SWI and the SWI plus NAM.

Furthermore, our confocal microscopy results also showed an elevated expression of p-JNK in the SWI mouse cortices. However, treatment using NAM, significantly regulated the expression level of active JNK, F _0.05(2,6)_ = 25.65; *P* < 0.01, while reducing the expression level in the SWI plus NAM-treated group (Figure 3A).

From these observations, we concluded that NAM protects brain damage, via regulation of RAGE signaling, and the reduction of oxidative stress and neuroinflammation in the injured mouse brains. The proposed mechanism of the neuroprotection of NAM treatment against brain injury-induced neurodegnerative conditions via regulation of RAGE/JNK/ NF-κB is explained in the Figure 1B.

### 3.2. NAM Inhibited Neuroinflammation and Reduced Lesion Volume in The Cortex of the Injured Mouse Brains 

Injuries to the brain evoked inflammatory responses and the activation of astrocytes and microglia [48,49]. Therefore, in the present study, using western blot analysis, we examined the activation of inflammatory mediators in the ipsilateral side of the treated mice, at day 7. Our results demonstrated a significant increase in the inflammatory mediators, such TNF-α, IL-1β, and NOS2, in the SWI mice group, compared to the saline-treated mice group. However, it was interesting to observe the inhibitory effects of NAM, while significantly reducing the levels of TNF-α, F _0.05(3,8)_ = 10.51 ; *P* < 0.05; IL-1β, F _0.05(3,8)_ = 5.753 ; *P* < 0.01 and NOS2 F _0.05(3,8)_ =7.579; *P* < 0.05 in the ipsilateral cortex of the brain-injured mice groups (Figure 3B).

**Figure 4:**
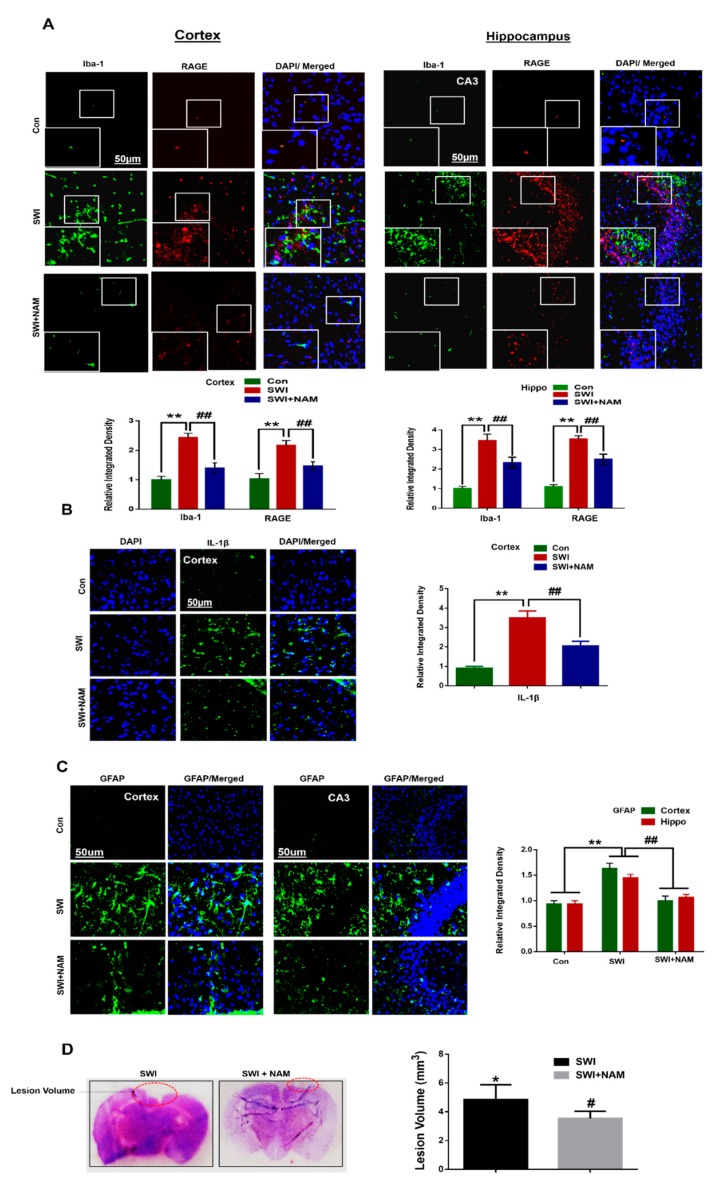
Effect of NAM on brain-injury-induced glial cells activation and lesion volume in the adult mice brain. (**A**) Shown are the representative immunofluorescence images among the treated groups. The squares represent the low (50 µm) and high magnification (30 µm) of the images. Double immunofluorescence reactivity of Iba-1 (marker for microglia) and RAGE, among the treated groups, represent the co-localization of the Iba-1 and RAGE in the cortex and in the hippocampal Cornu Ammonis 3 (CA3) region of the mouse brains. (**B**) Represents the immunoreactivity of the IL-1β among the treated groups. (**C**) Represents immunoreactivity of the GFAP (marker of active astrocytes) in the ipsilateral side of the injured mouse brains. (**D**) The lesion volume of the injured mouse brains and injured plus NAM-treated group. Values were calculated through ImageJ software. All values were taken from three independent experiments. Statistical analysis was done via one-way ANOVA, followed by post hoc analysis. *P* < 0.05 was considered to be statistically significant. The symbols * *P* < 0.05, and ** *P* < 0.01 represent significant differences between the control and SWI; # *P* < 0.05, and ## *P* < 0.01 represent significance differences between SWI and SWI plus NAM.

Our results followed the notion that NAM inhibits p-JNK and p-NF-κB in the AD mouse model [50,51]. Furthermore, we also observed the increased immunoreactivity of Iba-1 and RAGE, in the ipsilateral side of the cortex and CA3 region of the hippocampus of the SWI mice group, compared to the saline-treated mice group. Furthermore, we found an increased expression level of IL-1β in the ipsilateral cortex of the injured mouse brains. The confocal microscopy suggested that Iba-1 and RAGE were co-localized in the ipsilateral cortical region of the injured mouse brains. From these observations, we concluded that the activation of RAGE in the glial cell, might contribute to the deleterious effects on neuronal cells, following a brain injury. However, NAM treatment reduced the levels of Iba-1, F _0.05(2,3)_ = 118.4; *P* < 0.01; RAGE, F _0.05(2,3)_ = 117.5; *P* < 0.01 (Figure 4A), and IL-1β, F _0.05(2,6)_ = 27.46 ; *P <* 0.01 (Figure 4B), and it also inhibited the inflammatory response in the SWI mice group. A previous study reported that inhibition of active JNK, regulated the inflammatory response, and also triggered the glial cells [36]. Furthermore, we found that the immunoreactivity of GFAP (a marker of active astrocytes) showed the activation of astrocytes in the cortex and hippocampal CA3 (Cornu Ammonis 3) region of the injured mouse brains. Interestingly, NAM treatment significantly reduced the activation of astrocytes, while reducing GFAP, F _0.05(2,3)_ = 108.5; *P <* 0.01 in the SWI plus NAM-treated mice (Figure 4C). Blood brain barrier (BBB) and an enlarged lesion volume are the main indicators of brain injury. Therefore, we analyzed the lesion volume using cresyl violet staining [36]. Our results indicated that injuries to the brain increased the lesion volume and that NAM treatment reduced the lesion volume, which might probably regulate BBB break down (Figure 4D). From these observations, we concluded that treatment with NAM could inhibit neuroinflammation, probably via regulation of the cytokines infiltration in the injured brains, and by avoiding cell death.

#### 3.3. NAM Reduced Neuronal Apoptosis in TBI Mouse Brains

**Figure 5:**
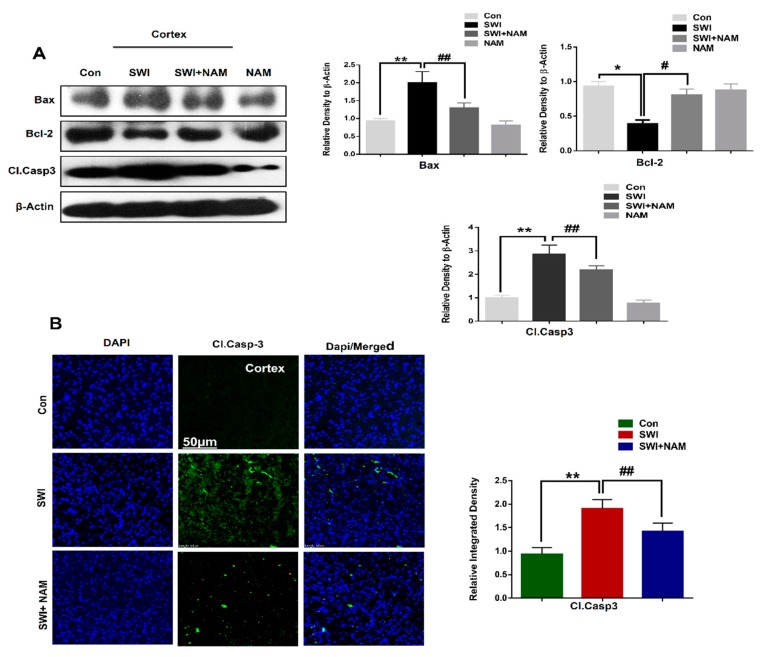
NAM treatment reduced the apoptotic neurodegeneration in brain-injured mice. (**A**) Western blot and histogram showing the expression level of apoptotic markers, such as cleaved caspase-3, Bax, and Bcl-2, in the brain-injured cortex alone and in the NAM-treated mice groups. The bands were quantified using Sigma Gel software. (**B**) Confocal microscopy images showing the caspase-3 immunoreactivity (Magnification X10). The caspase-3 fluorescence integrated density was analyzed with imageJ software. Values were calculated through ImageJ software. All values were taken from three independent experiments. Statistical analysis was done via one-way ANOVA, followed by post hoc analysis. *P* < 0.05 was considered to be statistically significant. The symbols * *P* < 0.05, and ** *P* < 0.01 represent significant differences between the control and SWI; # *P* < 0.05, and ## *P* < 0.01 represent significance differences between the SWI and SWI plus NAM.

Injuries to the brain caused intense apoptosis at the injury site. Similarly, our western blot results showed a significant increase in the expression level of pro-apoptotic markers, such as caspase-3 and Bax, with reduced anti-apoptotic marker expression, such as Bcl-2, in the ipsilateral cortex of the SWI mice group, compared to the saline-treated mice group, at day 7. Interestingly, NAM treatment reduced the expression of caspase-3, F _0.05(3,8)_ = 17.95; *P* < 0.05 and Bax, F _0.05(3,8)_ = 13.80; *P <* 0.01 and rescued the expression of Bcl-2, F _0.05(3,8)_ = 5.505; *P <* 0.05 in the injured mouse brains (Figure 5A). Furthermore, the results were validated through confocal microscopy analysis. Our immunofluorescence analysis indicated the elevated expression level of caspase-3 in the ipsilateral cortical region of the injured mouse brains. However, the caspase-3 expression F _0.05(3,8)_ = 27.18; *P <* 0.01 was reversed in the ipsilateral cortical region upon NAM treatment in the SWI plus NAM treated group (Figure 5B), suggesting strong anti-apoptotic effects of NAM in brain injuries. Recent studies have provided evidence that NAM treatment confers neuroprotection, via a reduction of the mitochondrial apoptotic pathway in the Aβ-treated animal brains [52]. 

**Figure 6:**
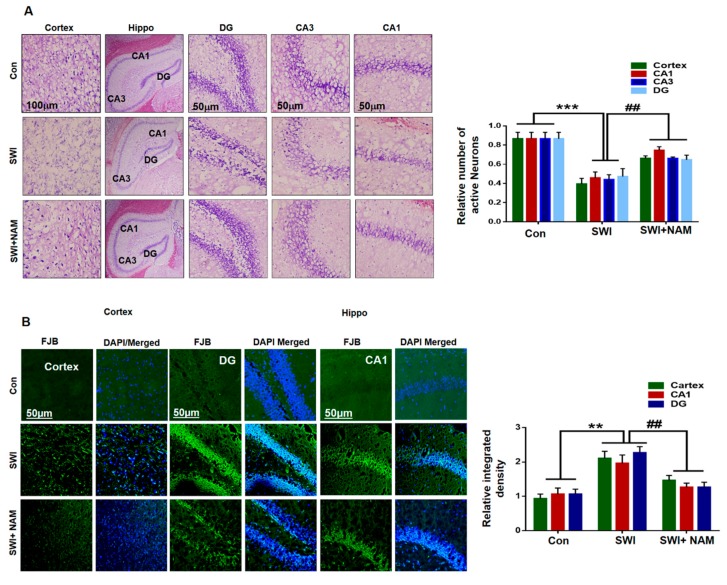
NAM reduced the injury-induced neuronal apoptosis in the mouse cortex. (**A**) Representatives images are the confocal microscopy images of Nissl staining in the cortex and hippocampal Cornu Ammonis 1 (CA1), CA3, and Dentate gyrus (DG) regions among the treated groups. (**B**) Confocal images of Fluoro-Jade B (FJB) +ve (dead) neuronal cells (Magnification X10). ImageJ software was used for images quantification. Values represent the mean ± SEM. All values were taken from three independent experiments. Statistical analysis was done through one-way ANOVA analysis. *P* < 0.05 was considered to be statistically significant. The symbols * *P* < 0.05, and ** *P* < 0.01, and *** *P* < 0.001 represent significant differences between the control and SWI; # *P* < 0.05, and ## *P* < 0.01 represent significance differences between SWI and SWI plus NAM.

Morphologically, the effect of NAM was investigated using Fluoro-Jade B (FJB) and Nissl staining in the cortex and the hippocampal regions. Nissl staining was performed to assess neuronal cell death in the ipsilateral cortex and hippocampus region of the injured mouse brain. Our results clearly indicated the reduced number of survival neurons in the cortex and CA1 (Cornu Ammonis 1), CA3 (Cornu Ammonis 3), and DG (Dentate gyrus) regions of the hippocampus of the injured mice brains, compared to the saline-treated normal mice brain. Similarly, NAM treatment reversed the effects and significantly increased the number of survival neurons in the SWI plus NAM-treated mice group; F _0.05(2,6)_ = 46.17; *P <* 0.001 Figure 6A. Furthermore, neuronal degeneration was confirmed through FJB staining. Our results clearly indicated an in increase in FJB+ve cells, which indicated an accelerated neuronal cell death in the cortex and the CA1, CA3, and DG regions of the hippocampus of the injured mice brains, compared to the saline-treated mouse group . Conversely, these effects were significantly reversed in the NAM-treated mouse groups; F _0.05(2,9)_ = 20.46; *P <* 0.01 (Figure 6B).

### 3.4. NAM Reversed the Synaptic Protein Loss and Improved Memory Dysfunction and Motor Neuronal Dysfunction

**Figure 7:**
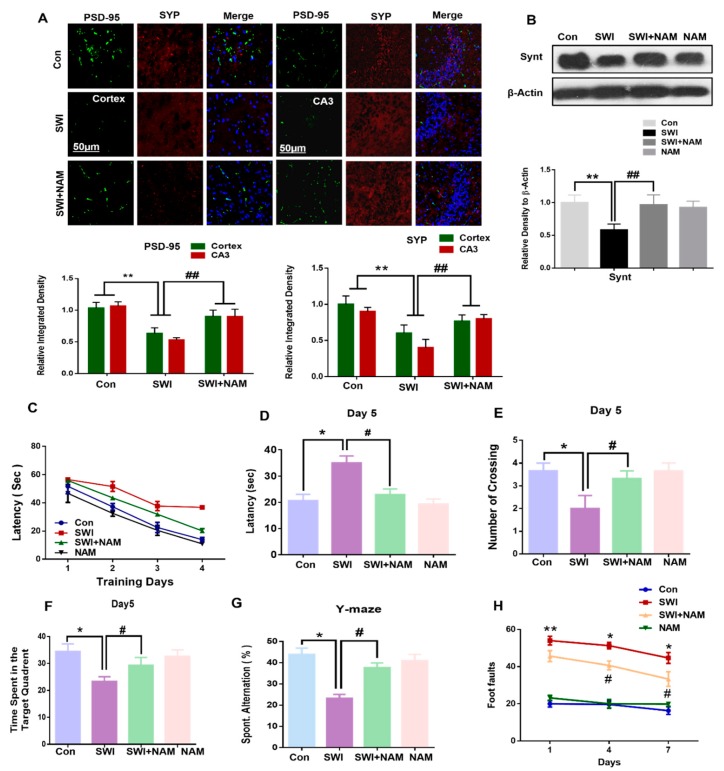
NAM reversed brain-injury-induced synaptic protein loss and memory impairment in the mice brain. (**A**) Confocal microscopy images represent the double immunofluorescence of PSD-95 and synaptophysin, among the treated groups. (**B**) Immunoblots and histogram represent the expression of synaptic protein syntaxin, among the treated groups. (**C,D**) The histogram represents latency to target platform, during training and during probe test, respectively. (**E**) Histogram showing the number of crossing during the probe test, on day 5. (**F**) Histogram of the time spent in the target quadrant during the probe test, on day 5. (**G**) Histogram showing the spontaneous alternation (in %) in the Y-maze behavior analysis. (**H**) Represents the foot faults at different time intervals, among the treated groups. The data are shown as a mean ± S.E.M. All values were taken from three independent experiments. Statistical analysis was done through one-way ANOVA analysis. *P* < 0.05 was considered to be statistically significant. The symbols * *P* < 0.05, and ** *P* < 0.01 represent significant differences between the control and SWI; # *P* < 0.05, and ## *P* < 0.01 represent significance differences between SWI and SWI plus NAM.

Synaptic dysfunction is a key feature of brain injury [53]. In our model, we examined the synaptic protein loss, using a western blot analysis. Our results demonstrated a significantly reduced level of syntaxin in the cortex of the injured mouse brains, compared to the saline-treated mice. However, the NAM treatment significantly reversed the deregulated expression levels of synaptic protein in the injured mouse brain, while increasing the expression level of the synaptic protein, the syntaxin, F _0.05(3,8)_ = 14.29; *P <* 0.01 in the SWI plus NAM-treated group. Similarly, the co-localization in the immunohistological results of PSD-95 and Synaptophysin (SYP), also revealed their reduced expression level in the cortex and hippocampus of the injured mouse brains. Likewise, NAM treatment rescued synaptic protein, PSD-95 (F _0.05(2,3)_=52.7; *P <* 0.01) and SYP (F _0.05(2,3)_ = 106.8; *P <* 0.01) loss, to the base level in the SWI mice (Figure 7A,B).

Additionally, the effect of NAM treatment on spatial learning and memory in injured mouse brains were evaluated using the Morris water maze (MWM), and Y-maze tests. Our MWM test revealed an increased escape latency to the target quadrant in the injured mouse brains, compared to the saline-treated mice. This validated the spatial learning deficits in the injured mouse brains. Interestingly, the NAM-treated mice group reduced the latency time, during a training session in the injured mouse brains (Figure 7C), which indicated that the NAM treatment improved memory function in the injured mice group. In parallel, the reported literature also observed the significant reversal potential of NAM treatment on cognitive abnormalities [51].

During the probe test, prolonged latency time to the target and the reduced number of crossings and time spent in the target quadrant were observed in the injured mouse brains. Inversely, NAM treatment reversed these abnormalities and significantly decreased the latency time, F _0.05(3,2)_ = 21.78; *P* < 0.05, increased the number of crossings, F _0.05(3,2)_ = 20.06; *P <* 0.05, as well as the time spent in the target quadrant, F _0.05(3,2)_ = 63.60; *P <* 0.05 (Figure 7D–F). The probe test further validated improved memory function in the mice that had received the NAM treatment. Next, we performed the Y-maze test and used the spontaneous alternation behavior (%) to analyze the spatial working memory. We observed that the mice that had received brain injury had a less spontaneous alternation behavior (in %), compared to the saline-treated mice (Figure 7G). However, we observed a significant increase in spontaneous alternation behavior (in %) in the NAM-treated mice, F _0.05(3,2)_ = 36.27; *P* < 0.05. Furthermore, we confirmed motor neuronal dysfunction via the beam walking test [36]. Our results of the beam walking test indicated a significant difference amongst the treatment groups (Figure 7H). However, we observed that the NAM treatment significantly reversed the increased motor neuronal abnormalities in the injured mice group. Overall, these results showed that NAM improved memory function, motor neuronal function, and behavioral outcomes in mice that had received an injury to the brain, with no major side effects. Previous studies have shown that activation of the RAGE signaling pathway is involved in the deterioration of memory functions and increases memory impairment.

## 4. Discussion

We developed a stab wound cortical injury in the animal model and explored several pathological conditions in the mouse brain, after 7 days. We further assessed the anti-oxidative and anti-inflammatory effects of a NAM treatment against brain-injury-induced neurodegenerative conditions in mice. Neuroinflammation and oxidative stress are the major hallmarks of brain injury [54]. RAGE activation is the major contributor to neuroinflammation and oxidative stress, as the activation of RAGE and NF-κB leads to the activation of several pro-inflammatory markers, which ultimately causes neuronal degeneration [55]. Previous studies have reported the activation of RAGE in the animal model of brain injury, as well as the activation of RAGE/NFKB signaling in the AD mouse model, which further trigger neuroinflammation in the brain [47,56]. Other studies have demonstrated the active role of RAGE in acting as an inflammatory mediator and inducer of oxidative stress, as well as driving the process of Aβ production and accumulation. RAGE is a multi-ligand receptor that can bind S-100 calcium-binding protein, Mac-1, mobility group protein (B) 1 (HMGB1), phosphatidylserine, and amyloid-β (Aβ) protein [57].

In the current study, we explored the increased expression levels of RAGE, active JNK, and p- NF-κB, in the ipsilateral side of the cortex and hippocampus, in the injured mouse brains. A recent study demonstrated the critical role of RAGE in detrimental cellular effects, including excitotoxicity and neurodegeneration [58]. Interestingly, NAM treatment inhibited these pathological markers in the ipsilateral cortex and hippocampus of injured mouse brains. Our results were consistent with previous results showing that NAM treatment increased the antioxidant enzymes and reduced the oxidative stress and amyloid precursor protein (Aβ generating protein) in Aβ injected mice [50]. Likewise, our immunohistological results revealed increased expression levels of active JNK in the cortical region of the injured mouse brains. Similarly, active JNK was significantly attenuated by NAM treatment, in the brains of the injured mice. Logically, from these results, we can hypothesize that the reversal effects of NAM treatment might be attributable to the reduction in ligand-based activation of RAGE, and that NAM might interfere with the ligand-based interaction of RAGE in the injured mice brain. Previous studies have demonstrated that NAM treatment reduced the level of Aβ in the AD mouse model [50].

We sought to investigate the anti-inflammatory effects of NAM treatment in the injured mouse brain. Previous literature has proven that brain injuries contribute to severe neuroinflammation, which ultimately leads to neuronal deterioration [59,60]. In the present study, we explored the inflammatory response, following brain injury in mice. Our immunohistological and immunoblot analyses showed that brain injury in mice evoked a remarkable increase in microglia and astrocytes-mediated inflammatory mediators in the ipsilateral side of the injured mouse brains. This inflammatory response might involve the activation of the RAGE/NF-κB signaling pathway, because previous studies have reported that the RAGE/NF-κB signaling pathway regulates inflammatory conditions [59]. In our study, NAM treatment inhibited the inflammatory response, while reducing the number of microglia and astrocytes, and it also suppressed the pro-inflammatory mediators in the ipsilateral cortex of the mice that received an injury to the brain. Previous reports have also demonstrated the protective effects of NAM treatment on neuroinflammation in the AD mouse model [52]. Recently, it was reported that NAM mononucleotide treatment inhibits neuroinflammation and neurodegeneration by inhibiting JNK in an AD animal model [50]. 

Neuronal apoptosis occurs through multiple cellular mechanisms, following secondary neuronal injury, after a primary brain injury [61]. It was found that RAGE activation induces inflammation, oxidative stress, and apoptosis via upregulation of NF-κB [62,63]. In the present study, we found that NAM inhibited apoptosis in an injured mouse brain model, while reducing the expression levels of caspase-3 and Bax and normalizing the expression of B-cl2, in the cortex of the SWI-treated mice. Several studies have identified the regulation of apoptosis with activation of RAGE/JNK, which causes cell death and neurodegeneration [64]. Another study reported that RAGE activation contributes to neuronal cell death in the animal model of AD [65]. Interestingly, our immunoreactivity results also showed a decreased level of caspase-3 in the ipsilateral cortical region of the injured mouse brains that received a NAM treatment, compared to the SWI group alone. Furthermore, confocal microscopy indicated an increase in FJB positive cells, which represented the increased number of dead neurons in the ipsilateral hippocampus and cortex regions of the injured mouse brains. Additionally, the neuronal density was elevated in the injured-brain, after NAM treatment at day 7. A previous study has demonstrated that NAM effectively reduced neuronal cell death after intracerebral hemorrhage [66]. These results showed the possible mechanism of a NAM neuroprotection, by regulating the RAGE/JNK/NF-κB signaling pathway in the injured mouse brains.

Synaptic loss originates with cognitive impairment and is a major hallmark of brain injuries [51,67]. To assess the synaptic protein level and memory impairment, we examined the expression level of synaptic markers and performed the Morris water maze test and Y-maze, respectively. For the expression levels of a synaptic protein marker, we analyzed the levels of synaptic proteins, such as syntaxin, using western blot. Interestingly, our results showed a decreased expression level of syntaxin in the injured mouse brain cortex, and it was significantly reversed to the baseline, in the injured mouse brains, upon NAM treatment. Furthermore, immunofluorescence analysis revealed decreased expression levels of synaptic proteins, such as PSD-95 and SYP, in the cortex and the hippocampus of the injured mice, which significantly increased to the baseline, in the NAM-treated injured-brain. The Morris water maze test was applied to analyze cognitive ability. Brain injuries contributes to cognitive issues, which is the major source of poor quality of life. The results revealed that brain injury induced cognitive dysfunction, motor dysfunction, and memory impairment on day 7. Our results were consistent with previous results, showing that NAM treatment reduced motor deficits [68,69]. A recent study demonstrated improvements in memory and learning, under the administration of NAM in TBI mouse [70]. The previous study also reported that NAM treatment significantly restored cognitive problems in an animal model of AD [71]. In the present study, we confirmed the effects of NAM and improved cognitive dysfunction, such as learning and memory, as well as motor dysfunction in the brain of the injured animal model. Further investigation is needed on the use of NAM treatment to reduce the RAGE levels and to study the possible mechanism of neuroprotection in animal models of several diseases like AD and PD, as well ischemia, stroke, and traumatic brain injury.

## 5. Conclusions 

This study provided evidence that brain injuries contribute to several pathological conditions in the brain. However, NAM treatment effectively ameliorated these conditions and protected neurodegenerative conditions, such as neuroinflammation, apoptosis, and synaptic dysfunction, via the regulation of RAGE/JNK/NF-κB, in an animal model of brain injury. Further study and a detailed mechanism of neuroprotection are required for several types of brain injury in animal models. The proposed mechanism of NAM neuroprotection against brain injury-induced neuro-inflammation, neuronal apoptosis, and memory impairment are summarized in Figure 1B.
